# OTUB1 stabilizes mismatch repair protein MSH2 by blocking ubiquitination

**DOI:** 10.1016/j.jbc.2021.100466

**Published:** 2021-02-26

**Authors:** Qiong Wu, Yaping Huang, Liya Gu, Zhijie Chang, Guo-Min Li

**Affiliations:** 1 Department of Basic Medical Sciences, Tsinghua University School of Medicine, Beijing, China; 2 Department of Radiation Oncology, University of Texas Southwestern Medical Center, Dallas, Texas, USA

**Keywords:** DNA mismatch repair, DNA damage, OTUB1, deubiquitination, mutability, protein stability, CHX, cycloheximide, KD, knocked down, MMR, mismatch repair, MNNG, N-methyl-N'-nitro-N-nitrosoguanidine

## Abstract

DNA mismatch repair (MMR) maintains genome stability primarily by correcting replication errors. MMR deficiency can lead to cancer development and bolsters cancer cell resistance to chemotherapy. However, recent studies have shown that checkpoint blockade therapy is effective in MMR-deficient cancers, thus the ability to identify cancer etiology would greatly benefit cancer treatment. MutS homolog 2 (MSH2) is an obligate subunit of mismatch recognition proteins MutSα (MSH2-MSH6) and MutSβ (MSH2-MSH3). Precise regulation of MSH2 is critical, as either over- or underexpression of MSH2 results in an increased mutation frequency. The mechanism by which cells maintain MSH2 proteostasis is unknown. Using functional ubiquitination and deubiquitination assays, we show that the ovarian tumor (OTU) family deubiquitinase ubiquitin aldehyde binding 1 (OTUB1) inhibits MSH2 ubiquitination by blocking the E2 ligase ubiquitin transfer activity. Depleting OTUB1 in cells promotes the ubiquitination and subsequent degradation of MSH2, leading to greater mutation frequency and cellular resistance to genotoxic agents, including the common chemotherapy agents N-methyl-N'-nitro-N-nitrosoguanidine and cisplatin. Taken together, our data identify OTUB1 as an important regulator of MSH2 stability and provide evidence that OTUB1 is a potential biomarker for cancer etiology and therapy.

DNA mismatch repair (MMR) is a highly conserved DNA repair pathway across species that plays a vital role in maintaining replication fidelity. The typical MMR reaction in eukaryotic cells involves mismatch recognition, mismatch removal, and repair DNA synthesis. Mismatch recognition is carried out by MutSα (MSH2-MSH6) or MutSβ (MSH2-MSH3), which triggers a series of downstream reactions including interacting with proliferating cell nuclear antigen and recruiting MutLα (MLH1-PMS2) and exonuclease 1 (Exo1) to a nearby strand break on the nascent DNA strand. Mismatch removal is conducted by Exo1, which excises the nascent strand from the nick up to and beyond the mismatch to generate a single-strand gap. Finally, DNA polymerase δ fills this gap, and the repair is concluded by nick ligation catalyzed by ligase I (1).

In addition to correcting replication errors, the MMR system also maintains genome stability by processing nonmismatch DNA lesions induced by genotoxic agents (1, 2). In this process, MutSα recognizes these DNA adducts to provoke strand-specific MMR (3, 4). However, because MMR is targeted to the newly synthesized strand and the adducts are located on the template strand, attempting to remove these DNA adducts causes a futile repair cycle (5), which induces apoptosis (2, 6). Thus, cells defective in MMR cannot sense DNA lesions and become highly resistant to genotoxic drugs (1, 7).

Consistent with its role in maintaining genome stability, defects in MMR due to gene mutations (1, 8, 9, 10), loss of expression by promoter hypermethylation (11, 12, 13), or abnormal protein modification/degradation (14, 15, 16) lead to a hypermutable phenotype and the development of various types of human cancer. Conversely, overexpression of key MMR factors, such as MSH2 and MLH1, results in an increased mutation frequency (17) or apoptotic cell death (18). Therefore, the precise regulation of the homeostasis of cellular MMR proteins is critical for maintaining genome stability.

The ubiquitination-proteosome system (UPS) regulates the protein proteostasis of a wide range of substrates under normal or stress conditions in human cells in a posttranslational manner. Previous studies have shown that the stability of MSH2 is regulated by HDAC6-mediated ubiquitination (16) and USP10-mediated deubiquitination (19). It has been noted that the rate of UPS-mediated MutSα degradation varies across different cell lines (20), and multiple motifs that can be ubiquitinated are present in MSH2, which implies that other ubiquitinases/deubiquitinases might also regulate MSH2 proteostasis. A recent mass spectrometry study of MMR protein interactomes revealed a potential interaction between MSH2 and OTUB1 (21), a deubiquitilating enzyme that belongs to the ovarian tumor (OTU) superfamily of predicted cysteine proteases (22, 23). This suggests that MSH2 could be a substrate of OTUB1. OTUB1 is a highly specific ubiquitin iso-peptidase that functions in two different ways. In the canonical way, OTUB1 acts as a cysteine protease to directly deubiquitinate its substrates (22). OTUB1 also works in a noncanonical way by interacting with E2 enzymes to inhibit the ubiquitin transfer to substrates that are involved in diverse biological pathways (24, 25).

Here, we demonstrate that OTUB1 interacts with MSH2 to prevent MSH2 from ubiquitination through the noncanonical way. Depleting OTUB1 impairs the cellular proteostasis of MutSα and leads to MMR deficiency–associated resistance to genotoxic agents. Therefore, this study has established OTUB1 as a novel regulator of MMR and DNA damage response.

## Results

### OTUB1 interacts with MSH2 *via* its deubiquitylation catalytic center

**Figure 1:**
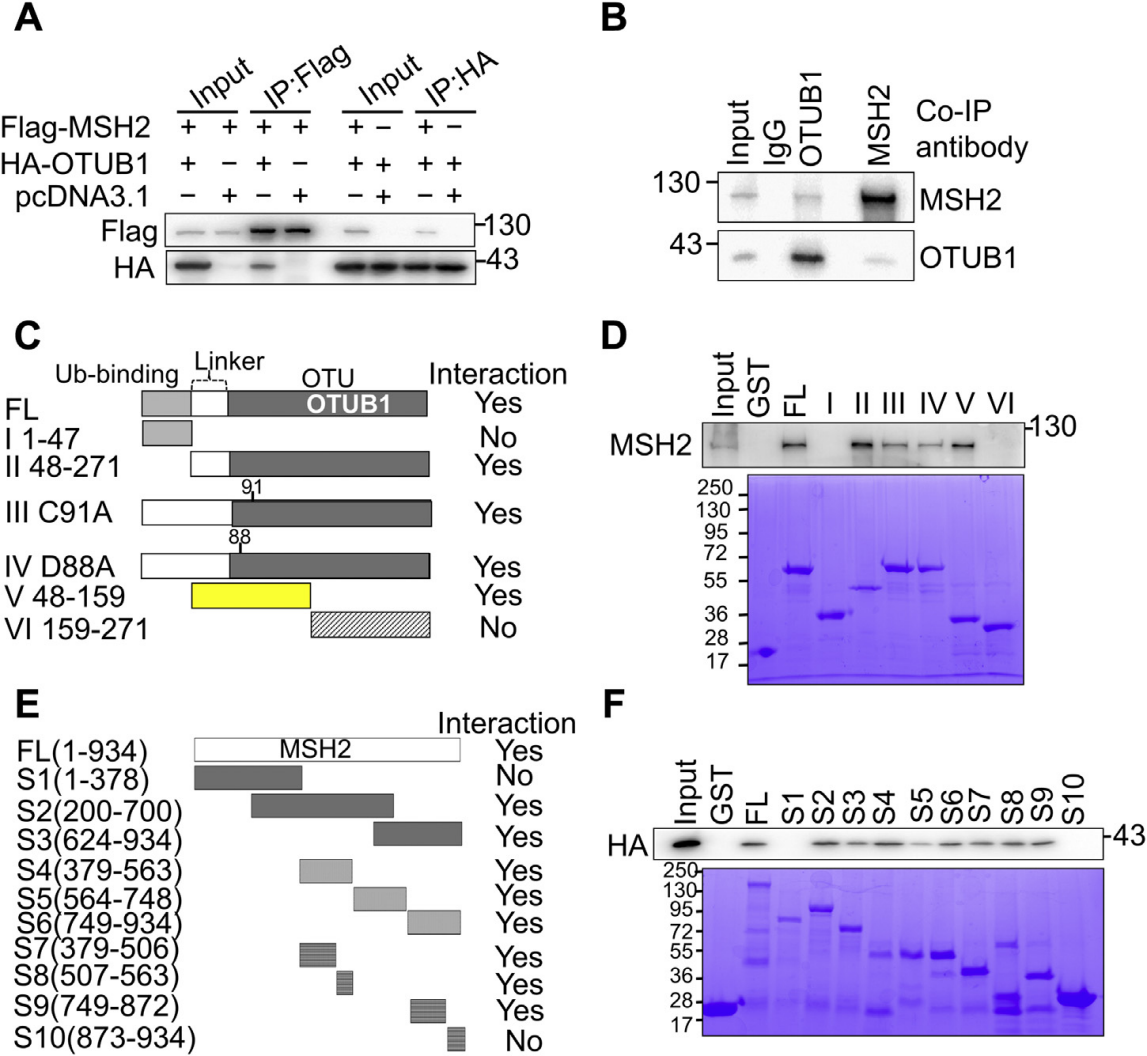
**OTUB1 interacts with MSH2.***A*, Flag-MSH2 and HA-OTUB1 were cotransfected into HEK293T cells, and the transfected cells were then treated with 20 μM MG132 for 6 h before harvesting. Exogenous co-immunoprecipitation (Co-IP) was performed with Flag agarose beads or HA magnetic beads to detect interactions between Flag-MSH2 and HA-OTUB1. *B*, Co-IP was performed with anti-OTUB1 and anti-MSH2 antibodies to detect the endogenous interaction between MSH2 and OTUB1 in HEK293T cells. *C*, diagram showing OTUB1 fragments used in the GST pulldown assay. *D*, western blot of MSH2 (*top*) that was pulled down by purified GST-OTUB1 and its truncated fragments (*bottom*). *E*, diagram of MSH2 constructs used in the GST pulldown assay. *F*, purified GST-MSH2 polypeptides (*bottom*) were incubated with cell lysates of HeLa cells overexpressing HA-OTUB1, and HA was blotted as indicated (*top*).

To determine whether OTUB1 interacts with MSH2, we expressed Flag-tagged MSH2 (Flag-MSH2) and HA-tagged OTUB1 (HA-OTUB1) in HEK293T cells and performed reciprocal pulldown experiments using Flag agarose and HA magnetic beads. We found that Flag-MSH2 and HA-OTUB1 pulled each other down (Fig. 1*A*), which suggests a specific interaction between MSH2 and OTUB1. We obtained similar results in a co-immunoprecipitation experiment to determine endogenous interactions between these two proteins by using an MSH2-specific or an OTUB1 antibody (Fig. 1*B*). Although the interaction between MSH2 and OTUB1 is relatively weak, it is much more specific than the control reactions in both the pulldown and the co-immunoprecipitation assays.

OTUB1 mainly contains three domains (Fig. 1*C*): the Ub-binding domain (1–47aa), the linker domain (48–85aa), and the OTU domain (85–271aa) (26). To determine which domain interacts with MSH2, we constructed GST-tagged full-length OTUB1 (FL) and truncated or residue-altered OTUB1s—OTUB1(1–47), OTUB1(48–271), OTUB1(48–159), OTUB1(159–271), OTUB1-C91A, and OTUB1-D88A (Fig. 1*C*)—and detected the interactions between these OTUB1 polypeptides and MSH2. GST pull-down assays showed that the FL OTUB1 strongly interacted with MSH2 (Fig. 1*D*). We also observed strong interactions between MSH2 and OTUB1(48–271) and OTUB1(48–159), but not between MSH2 and OTUB1(1–47) or OTUB1(159–271) (Fig. 1*D*). The deubiquitylation function of OTUB1 relies on the catalytic center containing amino acids C91, D88, and H265 (26), which could form a catalytic triad. To determine the role of this activity center in MSH2 deubiquitination, we measured MSH2’s interactions with the catalytic-dead mutants OTUB1-C91A and OTUB1-D88A. We found that both the C91A and D88A mutants could interact with MSH2 (Fig. 1*D*). Taken together, these results indicate that OTUB1 physically interacts with MSH2 *via* its middle C1 domain, including its catalytic center.

To determine which MSH2 domain interacts with OTUB1, we first generated GST-tagged full-length MSH2 and three GST-tagged MSH2 mutants: MSH2 (1–378), MSH2 (200–700), and MSH2 (624–934) (Fig. 1*E*), as described previously (19). We used these GST-tagged proteins to pull down HA-OTUB1 in HeLa cell lysates. As expected, FL-MSH2 could pull down HA-OTUB1 (Fig. 1*F*). Among the three mutants, MSH2 (200–700) and MSH2 (624–934) could precipitate OTUB1, which suggests that the ubiquitylated residues in MSH2 that are regulated by OTUB1 are located in the central and C-terminal domains. To narrow down the OTUB1 interaction domain(s), we further divided MSH2 fragments S2 and S3 into several smaller peptides (Fig. 1*E*, S4-S10) and performed the same pull-down assay. The results showed that all peptides except S10, which failed to pull down OTUB1, more or less interacted with OTUB1 (Fig. 1*F*). Therefore, we conclude that OTUB1 interacts with MSH2 mostly through the central domain.

### OTUB1 stabilizes MSH2

**Figure 2:**
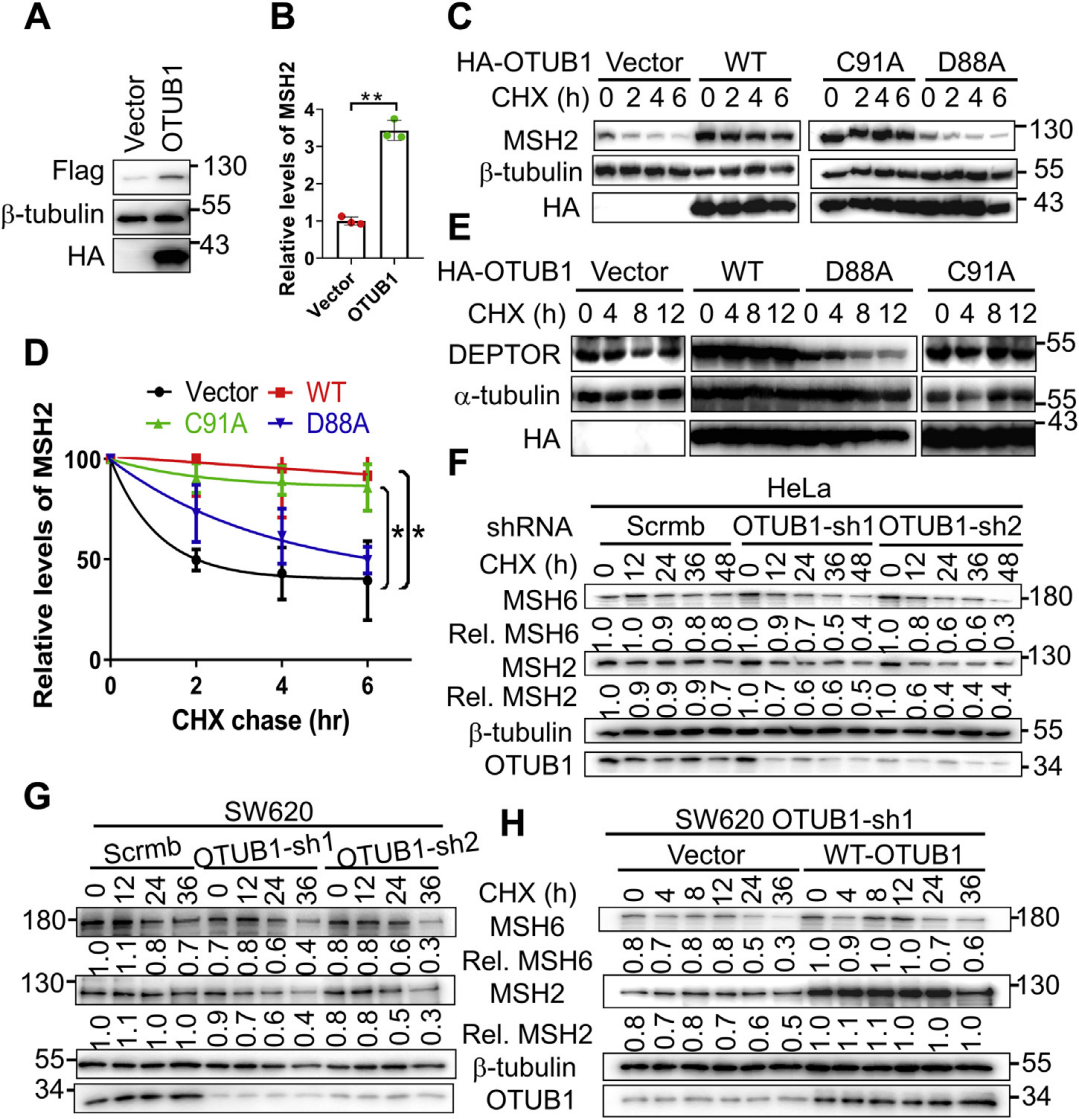
**OTUB1 stabilizes MSH2.***A*, the stability of Flag-MSH2 in the presence or absence of HA-OTUB1 was determined by western blot. *B*, quantification in three independent experiments of Flag-MSH2 levels in cells expressing HA-OTUB1, as shown in *A*. *C*, the effect of OTUB1 on the half-life of Flag-MSH2 was determined by CHX chase experiments. Flag-MSH2 and each of the OTUB1 constructs (WT, C91A and D88A) were cotransfected into HEK293T cells. Thirty-six hours later, the transfected cells were treated with CHX (50 μg/ml) and harvested at the indicated time points for western blotting analysis. *D*, quantification of the MSH2 half-life in three independent experiments, as shown in panel *B*. *E*, the effect of OTUB1 on the half-life of Flag-DEPTOR was determined by CHX chase experiments. The experiment was performed as in *C*. *F*–*H*, the half-life of MSH2 and MSH6 was determined similarly as in panel *B* in OTUB1-KD HeLa and SW620 cells, as well as in OTUB1-rescued SW620-KD cells. The relative expression levels of MSH2 or MSH6 are shown below the gel. The average of three repeats was used in statistical analyses (two-tailed *t* test). ∗ indicates *p* < 0.05, ∗∗ indicates *p* < 0.001.

To determine whether the OTUB1–MSH2 interaction regulates the stability of MSH2, we measured Flag-MSH2 levels in HEK293T cells with or without the overexpression of HA-OTUB1. We found that the Flag-MSH2 level was much higher in cells expressing HA-OTUB1 than in those without HA-OTUB1 expression (Fig. 2, *A* and *B*), which suggests that OTUB1 does indeed stabilize MSH2. To further explore this, we measured the half-life of MSH2 by treating cells with protein synthesis inhibitor cycloheximide (CHX). Consistently, the half-life of Flag-MSH2 was significantly higher in cells overexpressing OTUB1 (Fig. 2, *C* and *D*). Interestingly, the catalytic-dead mutant OTUB1 (C91A) also extended the half-life of Flag-MSH2; however, we did not observe MSH2 stabilization in cells expressing the OTUB1 (D88A) mutant (Fig. 2, *C* and *D*). Because the catalytic Cys91 and Asp88 are essential for the OTUB1 deubiquitination activity (27) and OTUB1’s inhibition of E2 ligases (24, 25), respectively, these results suggest that OTUB1 stabilizes MSH2 *via* noncanonical inhibition of E2 ligase activity. To verify this, we performed similar half-life analysis using a known OTUB1 noncanonical substrate, DEPTOR (27). The stability of Flag-DEPTOR was essentially the same as that of Flag-MSH2, *i.e.*, OTUB1 (C91A), but not OTUB1(D88A), could stabilize DEPTOR (Fig. 2*E*).

To further test whether OTUB1 is essential for maintaining MSH2 stability, we stably knocked down (KD) OTUB1 by two different small hairpin RNAs (shRNAs) in both HeLa and SW620 cells. We treated these cells with cycloheximide to inhibit the endogenous protein synthesis over a specific course of time and quantified the relative expression levels of MSH2 by western blot. The results showed that MSH2, as well as its partner MSH6, degraded much faster in OTUB1-KD cells than in control cells (Fig. 2, *F* and *G*). To rule out the off-target effects of shRNAs, we restored the expression of OTUB1 in OTUB1-KD cells and found that OTUB1 restoration indeed efficiently increased the half-life of both MSH2 and MSH6 in these cells (Fig. 2*H*). The observed stability correlation between MSH6 and MSH2 is consistent with the fact that MSH6 is unstable in the absence of MSH2 (28, 29, 30). However, whether MSH6’s stability is regulated independently by OTUB1 remains to be investigated. Nevertheless, the data presented here indicate that OTUB1 stabilizes MSH2 probably by inhibiting E2 activity.

### OTUB1 inhibits the ubiquitination of MSH2

**Figure 3:**
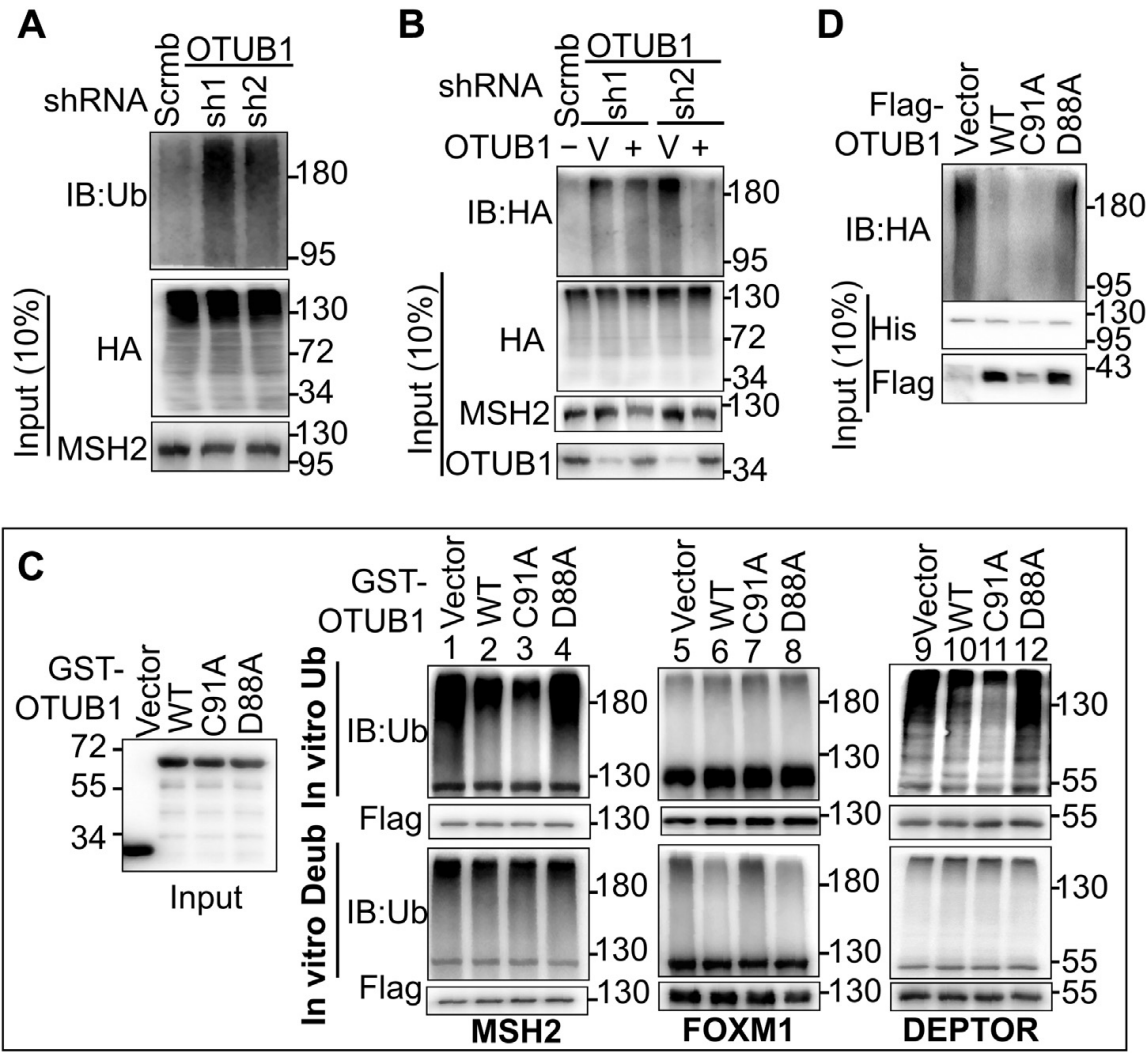
**OTUB1 inhibits MSH2 ubiquitination in a noncanonical way.***A*, analysis of MSH2 ubiquitination by denatured-IP in OTUB1-KD and control HeLa cells transfected with exogenous HA-Ub. Cell lysate was denatured and IP-ed with an MSH2 antibody, then examined by western blotting using an anti-Ub antibody. *B*, analysis of MSH2 ubiquitination by denatured-IP in OTUB1-KD HeLa cells with or without restoration of OTUB1 expression. V, empty vector. *C*, *in vitro* ubiquitination and deubiquitination assays to determine the mechanism by which OTUB1 regulates MSH2 stability. In the *in vitro* ubiquitination assay, individual Flag-tagged substrates (MSH2, FOXM1, and DEPTOR) were incubated with GST-OTUB1 (WT, C91A or D88A) and HA-Ub under the ubiquitination condition, as described in Experimental procedures. In the *in vitro* deubiquitination assay, individual preubiquitinated substrates (Flag-tagged MSH2, FOXM1 and DEPTOR) were incubated with GST-OTUB1 (WT, C91A or D88A) proteins under the deubiquitination condition, as described in Experimental procedures. Ubiquitination levels of MSH2 FOXM1 or DEPTOR were measured by an anti-Ub antibody. FOXM1 and DEPTOR are positive controls for direct deubiquitination and indirect deubiquitination by OTUB1, respectively. *D*, *in vivo* ubiquitination assay to determine the effects of WT, C91A, or D88A OTUB1 on MSH2 ubiquitination in HEK293T cells. His-MSH2, Flag-OTUB1 (WT, C91A or D88A), and HA-Ub were expressed in HEK293T cells, and His-MSH2 was pulled down using Ni-NTA agarose beads, as described in Experimental procedures. Ubiquitinated His-MSH2 was detected by western blotting using an HA antibody.

To test our hypothesis that OTUB1 stabilizes MSH2 by preventing its ubiquitination, we measured the ubiquitination levels of MSH2 in HeLa cells treated with MG132 and stably transfected with shRNA against OTUB1 or scrambled shRNA in the presence of HA-Ub. Under these conditions, ubiquitinated proteins are protected from degradation *via* the proteasome. Even though a similar amount of ubiquitinated proteins was present in all cells, significantly more ubiquitinated MSH2 was pulled down in OTUB1-KD cells (OTUB1-sh1 and OTUB1-sh2) than in cells with scrambled shRNA (Fig. 3*A*). Consistently, restoring OTUB1 to OTUB1-KD cells significantly reduced the ubiquitination level of MSH2 (Fig. 3*B*), as compared with cells transfected with an empty vector (V). Collectively, these results suggest that MSH2 is a substrate of OTUB1 and that OTUB1 inhibits MSH2 ubiquitination.

To further determine which OTUB1 function is involved in protecting MSH2 from being ubiquitinated, we performed *in vitro* assays to examine the individual OTUB1 proteins (WT, C91A, and D88A) for their ability either to directly deubiquitinate MSH2 or to inhibit MSH2 ubiquitination by E2 enzymes. The deubiquitination-specific substrate FOXM1 (31) and the noncanonical substrate DEPTOR were used as positive controls in these assays. In the *in vitro* ubiquitination assay, individual Flag-tagged substrates (MSH2, FOXM1, and DEPTOR) were incubated with GST-OTUB1 (WT, C91A, or D88A) and HA-Ub in a ubiquitination system containing rabbit reticulocyte lysate (RRL), as described previously (19), and ubiquitinated substrates were visualized by western blotting using a Ub antibody. As shown in Figure 3*C* (*in vitro* Ub), substrates MSH2 (lanes 1–4) and DEPTOR (lanes 9–12) exhibited much less ubiquitination in reactions containing WT OTUB1 (lanes 2 and 10) and OTUB1-C91A (lanes 3 and 11) than those containing OTUB1-D88A (lanes 4 and 12), but we observed little difference in ubiquitination among various OTUB1 proteins when the deubiquitination-specific substrate FOXM1 was used in the same assay (lanes 5–8, *in vitro* Ub). Conversely, the *in vitro* deubiquitination assays revealed that both OTUB1(WT) and OTUB1(D88A), but not OTUB1(C91A), could efficiently deubiquitinate FOXM1 (Fig. 3*C*, *in vitro* Deub, lanes 5–8), as expected. However, there was essentially no difference in ubiquitination removal among all reactions when MSH2 (lanes 1–4) and DEPTOR (lanes 9–12) were used in the deubiquitination assays; in other words, OTUB1 fails to remove established ubiquitination on MSH2 and noncanonical substrate DEOTOR. These results strongly suggest that OTUB1 blocks MSH2 ubiquitination by inhibiting the E2 ligase activity, rather than by directly deubiquitinating MSH2.

To further verify the *in vitro* ubiquitination and deubiquitination results, we expressed HA-Ub, His-MSH2, and three forms of Flag-OTUB1s (WT, C91A, and D88A) in HEK293T cells and performed His-tag pull-down assays, and we measured the ubiquitination levels of His-MSH2. We found less ubiquitinated MSH2 in HEK293T cells expressing both WT OTUB1 and OTUB1-C91A, but not OTUB1-D88A (Fig. 3*D*). These results agree with those depicted in Figure 3*C*. We therefore conclude that OTUB1 prevents MSH2 ubiquitination by suppressing the E2 ligase activity.

### OTUB1 depletion elevates mutation rate and resistance to genotoxic agents

MSH2 is an obligate subunit of both mismatch recognition proteins, MutSα and MutSβ, and depleting MSH2 leads to a mutator phenotype characterized by an elevated mutation frequency (1, 32). Since OTUB1 regulates MSH2 stability, we postulated that depleting OTUB1 would cause a mutator phenotype. To test this hypothesis, we performed HPRT assays to determine the spontaneous mutation frequency in WT and OTUB1-KD cells, as described previously (14). OTUB1 knockdown resulted in a 4- to 20-fold increase in mutation frequency in HeLa and SW620 cells, and restoring OTUB1 in OTUB1-KD cells rescued the mutator phenotype in both HeLa OTUB1-KD and SW620 OTUB1-KD cells (Table 1). Therefore, OTUB1’s regulation of MSH2 is critical to MMR and genome maintenance.

**Table 1:** Depletion of OTUB1 induces a mutator phenotype

Cell line		Mutation frequency (×10^7^)	Fold of increase in MF	*p* value
HeLa	Scramble	1.67 ± 0.02	1	
	OTUB1-sh1	6.83 ± 0.07	4.1	<0.01
	OTUB1-sh1+Vector	16.5 ± 0.1	11.6	<0.01
	OTUB1-sh1+OTUB1	1.99 ± 0.03	1.4	>0.05
SW620	Scramble	0.17 ± 0.08	1	
	OTUB1-sh2	4.6 ± 0.3	25.98	<0.01
	OTUB1-sh2+Vector	3.63 ± 0.13	20.47	<0.01
	OTUB1-sh2+OTUB1	0.28 ± 0.03	1.59	>0.05

**Figure 4:**
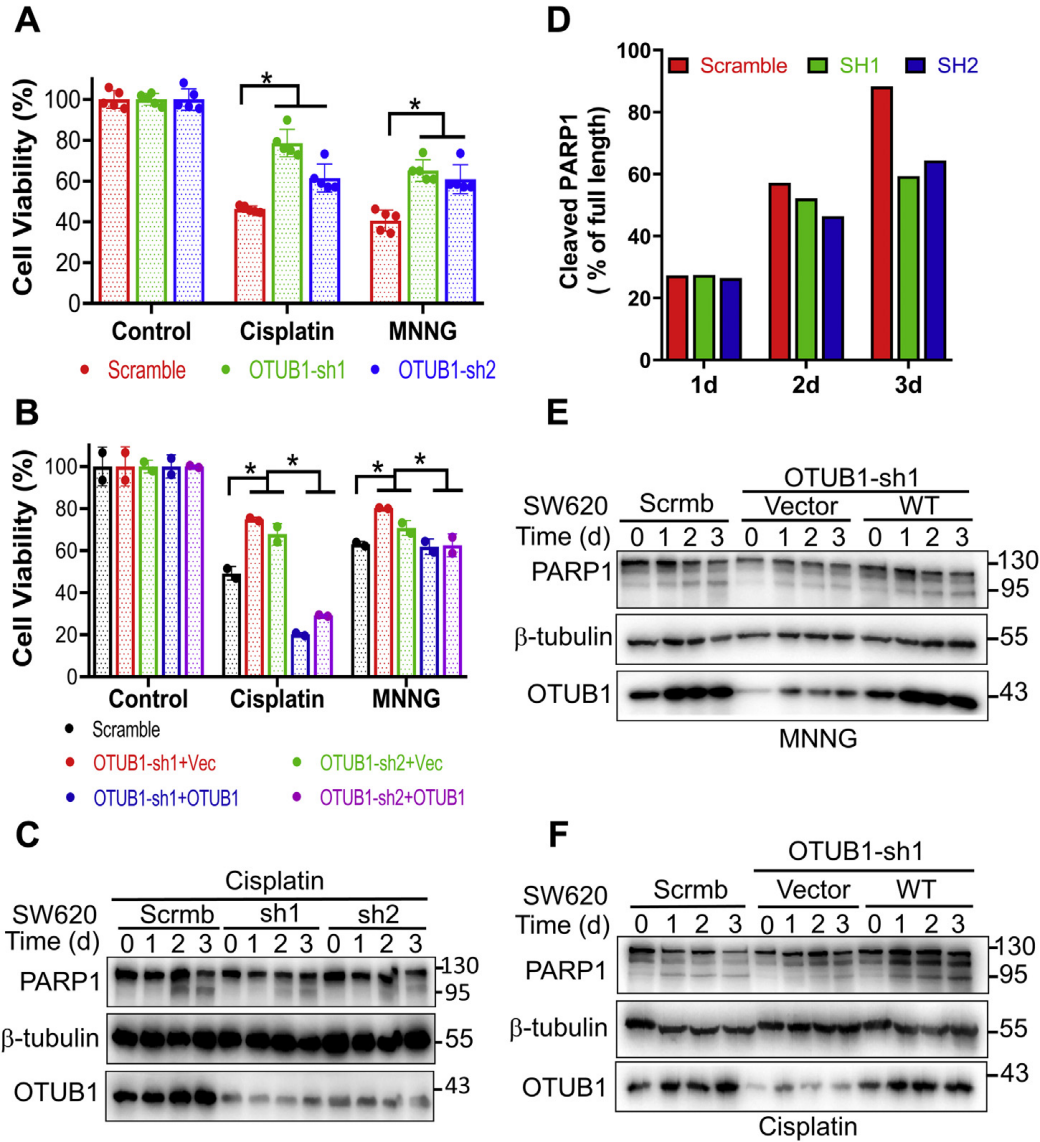
**Depletion of OTUB1 confers cellular resistance to genotoxic agents.***A* and *B*, cell viability analyses of OTUB1-KD (sh1 and sh2), OTUB1-rescued (+OTUB1), and control (scrambled) SW620 cells treated without (control) or with cisplatin or MNNG, as indicated, using Cell Counting Kit-8 assay. *C*, detection of cleaved PARP1 by western blotting in OTUB1-KD and control SW620 cells treated with cisplatin for the indicated times. *D*, quantification of cleaved PARP1 (% of the full-length PARP1) shown in *C*. *E* and *F*, detection of cleaved PARP1 in response to MNNG (*E*) and cisplatin (*F*) treatment by western blotting in OTUB1-KD SW620 cells rescued with OTUB1 for the indicated times. The average of five (panel *A*) or two (panel *B*) repeats was used in statistical analyses (two-tailed *t* test). ∗ indicates *p* < 0.05.

MMR proteins, particularly MutSα, also play important roles in DNA damage signaling, which leads to apoptotic cell death (2). Thus, defects in MSH2 render cells tolerant to many genotoxic agents, such as N-methyl-N'-nitro-N-nitrosoguanidine (MNNG) and cisplatin (1, 2). To test whether OTUB1 deficiency compromises MMR’s role in DNA damage signaling and promotes drug tolerance, we treated control and OTUB1-KD SW620 cells with MNNG and cisplatin and measured their survival after treatment. We found that OTUB1-KD cell lines, whether OTUB1-shRNA1 or OTUB1-shRNA2, were more tolerant of cisplatin or MNNG treatment than control SW620 cells (Fig. 4*A*). To rule out potential off-target effects in the knockdown cells, we performed the survival assay in OTUB1-restored SW620-OTUB1-KD cells. The results showed that restoring OTUB1 expression to SW620-OTUB1-KD also restored drug sensitivity (Fig. 4*B*), which confirms that OTUB1 is involved in DNA damage–induced response, likely by stabilizing cellular MSH2.

To determine whether SW620 cells undergo apoptotic cell death in response to treatment, we measured the cleavage of poly(ADP-ribose) polymerase 1 (PARP1), a hallmark of apoptosis (33, 34). The levels of cleaved PARP1 increased in a time-dependent manner in both control and OTUB1-KD SW620 cells treated with cisplatin (Fig. 4*C*). We quantified the PARP1 cleavage as previously described (35), and the results showed that the cleaved PARP1 level in OTUB1-KD cells was lower than in control cells (Fig. 4*D*). We performed the same analysis in OTUB1-rescued OTUB1-KD cells and found that restoring the expression of OTUB1 in OTUB1-KD cells resensitized cells to genotoxic agents including MNNG and cisplatin (Fig. 4, *E* and *F*). Similar results were also observed in HeLa and OTUB1-KD HeLa cells (Fig. S1). Collectively, these observations support the hypothesis that OTUB1 regulates genomic stability and cellular response to genotoxic agents by controlling MSH2 stability.

## Discussion

MMR is a critical genome maintenance system. Previous studies have shown that both loss and overexpression of key MMR components such as MutSα and MutLα lead to a mutator phenotype and predispose to cancers (1, 32, 36). Thus, maintaining MSH2 at an appropriate cellular level is essential. This is indeed the case, as the expression and stability of MSH2, as well as other MMR components, are precisely regulated in human cells *via* transcriptional and posttranscriptional mechanisms (12, 37, 38, 39, 40). Emerging evidence suggests that the stability of MSH2, an obligate subunit of MutSα (MSH2-MSH6) and MutSβ (MSH2-MSH3), is also tightly regulated posttranslationally (16, 19). Here, we identified OTUB1 as a novel regulator of MSH2, likely by inhibiting E2 ligase activity to prevent MSH2 ubiquitination. Depleting OTUB1 or impairing its noncanonical function that inhibits E2 activity enhances MSH2 ubiquitination (Fig. 3) and subsequent degradation *via* the proteasome (Fig. 2). This leads to a mutator phenotype (Table 1) and cellular resistance to certain genotoxic agents (Fig. 4). Given the diverse implications of MMR in cancer development and therapy (41, 42, 43), OTUB1 could be a potential biomarker for diagnosis and intervention in MMR-associated cancers.

We show that OTUB1 interacts with the central domain of MSH2 (Fig. 1, *E* and *F*). This suggests that OTUB1 stabilizes MSH2 by inhibiting MSH2 ubiquitination in the central domain, which overlaps with the HDAC6 interaction domain on MSH2 (16). It is possible that OTUB1 prevents MSH2 ubiquitination by inhibiting the E2 activity of HDAC6 or by competing with HDAC6 for the MSH2 residues located in the central domain. Another deubiquitinase, USP10, has been reported to interact with and deubiquitinate MSH2 in the N terminus (19), which implies that USP10 and OTUB1 may work nonredundantly to regulate the stability of MSH2 in human cells. This may also explain why MSH2’s half-life is very long even in cells depleted of either OTUB1 (Fig. 2) or USP10 (19). MutSα, as an important MMR factor, is known to be fairly stable during the whole course of cell division (14). Thus, OTUB1 and USP10 may play critical roles in maintaining the stability of MSH2 throughout the cell cycle. Nevertheless, this possibility has to be confirmed by future studies.

Previous studies have implicated OTUB1 in DNA damage signaling (24, 25, 44). However, the mechanism by which OTUB1 is involved in this process is not fully understood. We show here that OTUB1 regulates MMR-triggered DNA damage signaling by preventing MSH2’s ubiquitination. Thus, we believe that OTUB1 is likely a key regulator of proteins involved in DNA damage response, including those required for sensing and repairing replication errors and DNA strand breaks (24, 25, 44). As it does in regulating MSH2, OTUB1 may stabilize other DNA damage signaling factors by inhibiting the E2 ubiquitin ligase activity in a noncanonical manner. This regulation of DNA damage signaling factors in genome stability underscores OTUB1’s importance in cancer susceptibility and therapy. Because tumors defective in MMR exhibit high responsiveness to checkpoint blockade immunotherapy (42, 43), and because DNA damage response factors, particularly those involved in double-strand break repair, influence radiation and chemotherapy (45, 46), understanding the molecular mechanisms by which OTUB1 precisely regulates individual DNA damage response pathways will significantly benefit cancer treatments.

## Experimental procedures

### Cell culture and transfection

HeLa and HEK293T cells were grown in Dulbecco’s modified Eagle’s medium supplemented with 5% NBS and 5% FBS, penicillin (100 U/ml), and streptomycin (100 g/ml). SW620 cells were grown in RMPI1640 medium with the same supplements. All cells were maintained in a 37 °C incubator with 5% CO_2_. Hieff Trans Liposomal Transfection Reagent (Yeasen, 40802ES02) was used for transfection according to the manufacturer’s protocol.

### Plasmids, antibodies, reagents, and chemicals

We used pcDNA3.1 (+) vector to construct plasmids transiently expressing HA/Flag/His-tagged wild-type (WT) and mutant OTUB1 and MSH2. PLEX-MCS was used to construct the HA-OTUB1 plasmid for establishing the OTUB1-overexpressed HeLa cell line. PLVX-IRES-ZsGreen1 was used to restore OTUB1 expression in OTUB1 KD cells. pRK5-HA-ubquitin plasmid was transiently transfected for denatured immunoprecipitation (IP) and *in vivo* ubiquitination assay, and pGEX-4T-1 was used to construct bacterial expression plasmids, including GST-MSH2, GST-MSH2-S1(1–378), GST-MSH2-S2(200–700), GST-MSH2-S3(624–934), GST-MSH2-S4(379–563), GST-MSH2-S5(564–748), GST-MSH2-S6(749–934), GST-MSH2-S7(379–506), GST-MSH2-S8(507–563), GST-MSH2-S9(749–872), GST-MSH2-S10(873–934), GST-OTUB1, GST-OTUB1-N-terminus (1–47), GST-OTUB1-OTU domain(48–271), GST-OTUB1-C91A, GST-OTUB1-D88A, GST-OTUB1-C1(1–159), and GST-OTUB1-C2(160–271). In addition, pLKO.1 was used to construct shRNA plasmid to knock down OTUB1. psPAX2 and pMD2.G were used to pack virus.

We used the following antibodies in this study: anti-OTUB1 (Abcam ab175200, Sigma O9764), anti-MSH2 (CST 2017S, BD Biosciences 556349), anti-β-tubulin (Santa Cruz sc-166729), anti-Flag (Yeasen 30503ES60), anti-HA (Yeasen 30701ES60), anti-GST (Yeasen 30901ES50), anti-PARP1 (CST 9532S), anti-ubiquitin (CST 3936S), anti-His (CST 2366S) and anti-IgG (Rabbit) isotype control (CST 3900), anti-MSH6 (Abcam 92471), anti-α-tubulin (Easybio BE0031). For IP and denatured-IP, primary antibodies were diluted at a ratio of 1:100. For western blotting, anti-α-tubulin, anti-GST, and anti-HA antibodies were diluted at a ratio of 1:3000, and all other antibodies were diluted at a ratio of 1:1000.

6-Thioguanine (A4882), MG132 (C2211), ATP (A2383), L-Glutathione reduced (G4251) and DTT (D0632) were purchased from Sigma. Puromycin (S7417), Cycloheximide (1758–9310), and cisplatin (HZB0054-100) were purchased from Sellect, INALCO, and HARVEY, respectively. Anti-Flag Affinity Gel (B23101), anti-HA magnetic beads (B26202), and protein A/G magnetic beads (B23202) were from Bimake. GSTSep Glutathione agarose resin (20507ES50), Hisep Ni-NTA agarose resin (20503ES10), and Cell Counting Kit (40203ES60) were purchased from Yeasen. AnnexinV Alexa Fluor488/PI apoptosis detection kit (FXP022) was purchased from 4A Biotech. HA-UB purified protein (U-110) and Rabbit reticulocyte lysate (RRL) were purchased from Boston Biochem and Promega, respectively.

### Establishing OTUB1 knockdown and overexpressed stable cell lines

HEK293T cells were transfected with the target plasmid, packaging plasmids psPAX2 and pMD2.G, at a 4:3:1 ratio by using the liposome transfection agent. Seventy-two hours later, the medium supernatants were collected and filtered by using a 0.45-nm filter. The viruses were then immediately added into culture medium of targeted cells. Twenty-four hours postinfection, cells were cultured for another 24 h in fresh medium. Then, the cells were selected by 10 μg/ml puromycin for about 2 weeks until the uninfected control cells were all dead. We collected the surviving cells to determine the gene editing efficiency by western blotting using an anti-OTUB1 antibody.

### Immunoprecipitation and western blotting

Cells were harvested and lysed in lysis buffer (50 mM Tris-HCl, PH 8.0, 150 mM NaCl, 1 mM EDTA, 1% NP-40) for 30 min on ice, then centrifuged at 12,000 rpm at 4 °C for 10 min. Then, the soluble supernatant was incubated with protein A/G beads for 2 h to preclear nonspecific binding proteins, which was followed by incubation with the primary antibody or isotype control overnight at 4 °C. Protein A/G beads were added and incubated for another 3 h at 4 °C to pull down the antibody–protein complex. When performing IP with Flag or HA beads, the cell lysate was incubated with beads directly at 4 °C overnight. All immunoprecipitated beads were washed several times with cell lysis buffer before they were denatured for western blotting.

### GST pull-down assay

Bacterial BL21 cells transformed with a target GST plasmid were grown to the log phase (OD∼0.8), then the expression of a GST-tagged fusion protein was induced by isopropyl-thio-β-D-galactoside (IPTG) overnight at 16 °C. The collected cells were sonicated in STE buffer (10 mM Tris-HCl pH 8.0, 150 mM NaCl, 1 mM EDTA, 5 mM dithiothreitol, 1% Triton X-100 [w/v]) until the liquid turned clear. The soluble GST-tagged fusion proteins were purified *via* glutathione-agarose beads in the presence of 3% (w/v) Triton X-100. The purified protein on beads was incubated with HeLa or OTUB1 overexpressed HeLa cell lysates overnight at 4 °C to pull down the interacting proteins.

### Cycloheximide (CHX) chase experiment

To determine the half-life of endogenous MSH2, we seeded equal numbers of cells into 6-well plates, then changed the medium with fresh medium containing 20 μg/ml CHX. Cells were harvested for western blotting at the indicated time points. To determine the half-life of the exogenous MSH2 in the presence of WT and mutant OTUB1, HEK293T cells were cotransfected with plasmid carrying Flag-MSH2 and HA-OTUB1 (WT, C91A and D88A) for 36 h and treated with 50 μg/ml CHX for the indicated times before western blotting.

### Denatured IP

The denatured IP experiment was performed as described (47). Briefly, cells cultured in the 6-well plates were transfected with 1 μg HA-UB plasmids for 48 h, then treated with 20 μM MG132 for 6 h to inhibit proteosomal degradation. Cells were harvested and lysed in the denatured lysis buffer (20 mM Tris-HCl, pH 8.0, 1% SDS), and the cell lysate was boiled at 95 °C for 15 min. The denatured lysate was then diluted 10 times with denatured IP buffer (10 mM Tris-HCl pH 8.0, 150 mM NaCl, 1% Trition 100, 1 mM EDTA), followed by immunoprecipitation. Finally, the immunoprecipitated beads were washed four times with washing buffer (20 mM Tris pH 8.0, 150 mM NaCl).

### *In vivo* deubiquitination assay

The HA-UB, His-MSH2 and OTUB1 (WT, C91A, or D88A) plasmids were cotransfected into HEK293T cells for 2 days, and the cells were then treated with 20 μM MG132 for 6 h before harvesting. Cells were then lysed in Buffer A (6 M guanidine-HCl, 0.1 M Na_2_HPO_4_/NaH_2_PO_4_, 0.01 M Tris-HCl pH 8.0, 10 mM imidazole, 10 mM β-mecaptoethanol). The resulting cell lysates were incubated with Ni-NTA agarose beads overnight at room temperature, and the beads were then washed with Buffer A, Buffer B (8 M urea, 0.1 M Na_2_HPO_4_/NaH_2_PO_4_, 0.01 M Tris-HCl pH 8.0, 10 mM β-mercaptoethanol), Buffer C (8 M urea, 0.1 M Na2HPO4/NaH_2_PO4, 0.01 M Tris-HCl pH 6.3, 10 mM β-mercaptoethanol), and Buffer D (Buffer C with 0.1% Tween-20). The proteins on the beads were denatured for western blot analysis.

### *In vitro* ubiquitination and deubiquitination assays

The Flag-MSH2 protein was purified from HEK293T cells overexpressing Flag-MSH2 using anti-Flag affinity agarose beads. GST and GST-OTUB1 (WT, C91A, or D88A) were purified using GST glutathione agarose beads. For *in vitro* ubiquitination assay, the Flag beads were incubated with the same amount of GST or a GST-OTUB1 protein (WT, C91A, or D88A) in the presence of HA-UB, rabbit reticulocyte lysate (RRL), and 5 mM ATP in the ubiquitination buffer (50 mM Tris-HCl pH 7.5, 100 mM NaCl, 2.5 mM MgCl_2_, and 1 mM DTT) for 2 h at 37 °C. After washing with PBST, the protein samples were separated by SDS PAGE gels, and ubiquitinated proteins were detected by western blotting using an antibody against Ub, as previously described (19). For *in vitro* deubiquitination assays, preubiquitinated substrates on Flag-beads were washed with PBST three times, followed by deubiquitination buffer (50 mM Tris-HCl pH 8.0, 50 mM NaCl, 5 mM MgCl_2_, 1 mM ATP, 1 mM EDTA, 10 mM DTT, 5% glycerol) once. The Flag-beads were incubated with the same amount of GST or GST-OTUB1 (WT, C91A, or D88A) in deubiquitination buffer at 37 °C for 2 h, followed by SDS PAGE and western blotting analyses to detect the levels of ubiquitination, as described previously (19).

### Drug treatment and apoptosis analysis

The same numbers of cells were seeded in 6-well plates and treated with 15 μM 6-thioguanine (6-TG) for 24 h, or 10 μM MNNG) or 10 μM cisplatin for 1 h. The cells were rinsed twice with fresh medium and cultured for another 3 days. All cells, including the suspended ones, were collected, and half of them were used for AnnxienV/PI staining according to the manufacturer’s recommendation. The stained cells were then analyzed by flow cytometry. For the PARP1 assay, the treatment procedures were similar to those described above, except that apoptotic death was determined by PARP1 cleavage *via* western blotting assay. Cell viability assay was performed using the Cell Counting Kit-8 (CCK-8) reagent as instructed by manufacturer’s protocol.

### HPRT assay

For each cell line, two replicates of 5 × 10^5^ cells were plated in a 100 mm dish and treated with 6-TG (15 μM) for 4 days, then the cells were cultured in fresh medium for another 2 weeks. Meanwhile, each cell line’s colony formation ability was determined by seeding two replicates of 500 cells without 6-TG treatment. The colonies were stained with 0.5% crystal violet. The mutation frequency was calculated by dividing the number of surviving colonies in the 6-TG treated groups by the number of total seeded cells while normalized to the colony formation ability, as described (14).

## Data availability

All the data described in the article are within the article.

## Supporting information

This article contains supporting information.

## Conflict of interest

The authors declare that they have no conflicts of interest with the contents of this article.

## References

[1] Li G.M. (2008). Mechanisms and functions of DNA mismatch repair. Cell Res..

[2] Li G.M. (1999). The role of mismatch repair in DNA damage-induced apoptosis. Oncol. Res..

[3] Duckett D.R., Drummond J.T., Murchie A.I., Reardon J.T., Sancar A., Lilley D.M., Modrich P. (1996). Human MutSalpha recognizes damaged DNA base pairs containing O6-methylguanine, O4-methylthymine, or the cisplatin-d(GpG) adduct. Proc. Natl. Acad. Sci. U. S. A..

[4] Li G.-M., Wang H., Romano L.J. (1996). Human MutSa specifically binds to DNA containing aminofluorene and acetylaminofluorene adducts. J. Biol. Chem..

[5] York S.J., Modrich P. (2006). Mismatch repair-dependent iterative excision at irreparable O6-methylguanine lesions in human nuclear extracts. J. Biol. Chem..

[6] Wu J., Gu L., Wang H., Geacintov N.E., Li G.-M. (1999). Mismatch repair processing of carcinogen-DNA adducts triggers apoptosis. Mol. Cell. Biol..

[7] Karran P. (2001). Mechanisms of tolerance to DNA damaging therapeutic drugs. Carcinogenesis.

[8] Kinzler K.W., Vogelstein B. (1996). Lessons from hereditary colorectal cancer. Cell.

[9] Kolodner R.D., Marsischky G.T. (1999). Eukaryotic DNA mismatch repair. Curr. Opin. Genet. Dev..

[10] Kunkel T.A., Erie D.A. (2005). DNA mismatch repair. Annu. Rev. Biochem..

[11] Herman J.G., Umar A., Polyak K., Graff J.R., Ahuja N., Issa J.P., Markowitz S., Willson J.K., Hamilton S.R., Kinzler K.W., Kane M.F., Kolodner R.D., Vogelstein B., Kunkel T.A., Baylin S.B. (1998). Incidence and functional consequences of hMLH1 promoter hypermethylation in colorectal carcinoma. Proc. Natl. Acad. Sci. U. S. A..

[12] Kane M.F., Loda M., Gaida G.M., Lipman J., Mishra R., Goldman H., Jessup J.M., Kolodner R. (1997). Methylation of the hMLH1 promoter correlates with lack of expression of hMLH1 in sporadic colon tumors and mismatch repair-defective human tumor cell lines. Cancer Res..

[13] Veigl M.L., Kasturi L., Olechnowicz J., Ma A.H., Lutterbaugh J.D., Periyasamy S., Li G.M., Drummond J., Modrich P.L., Sedwick W.D., Markowitz S.D. (1998). Biallelic inactivation of hMLH1 by epigenetic gene silencing, a novel mechanism causing human MSI cancers. Proc. Natl. Acad. Sci. U. S. A..

[14] Li F., Mao G., Tong D., Huang J., Gu L., Yang W., Li G.M. (2013). The histone mark H3K36me3 regulates human DNA mismatch repair through its interaction with MutSalpha. Cell.

[15] Ortega J., Li J.Y., Lee S., Tong D., Gu L., Li G.M. (2015). Phosphorylation of PCNA by EGFR inhibits mismatch repair and promotes misincorporation during DNA synthesis. Proc. Natl. Acad. Sci. U. S. A..

[16] Zhang M., Xiang S., Joo H.Y., Wang L., Williams K.A., Liu W., Hu C., Tong D., Haakenson J., Wang C., Zhang S., Pavlovicz R.E., Jones A., Schmidt K.H., Tang J. (2014). HDAC6 deacetylates and ubiquitinates MSH2 to maintain proper levels of MutSalpha. Mol. Cell.

[17] Shcherbakova P.V., Kunkel T.A. (1999). Mutator phenotypes conferred by MLH1 overexpression and by heterozygosity for mlh1 mutations. Mol. Cell. Biol..

[18] Zhang H., Richards B., Wilson T., Lloyd M., Cranston A., Thorburn A., Fishel R., Meuth M. (1999). Apoptosis induced by overexpression of hMSH2 or hMLH1. Cancer Res..

[19] Zhang M., Hu C., Tong D., Xiang S., Williams K., Bai W., Li G.M., Bepler G., Zhang X. (2016). Ubiquitin-specific peptidase 10 (USP10) deubiquitinates and stabilizes MutS homolog 2 (MSH2) to regulate cellular sensitivity to DNA damage. J. Biol. Chem..

[20] Hernandez-Pigeon H., Laurent G., Humbert O., Salles B., Lautier D. (2004). Degadration of mismatch repair hMutSalpha heterodimer by the ubiquitin-proteasome pathway. FEBS Lett..

[21] Chen Z., Tran M., Tang M., Wang W., Gong Z., Chen J. (2016). Proteomic analysis reveals a novel mutator S (MutS) partner involved in mismatch repair pathway. Mol. Cell. Proteomics.

[22] Balakirev M.Y., Tcherniuk S.O., Jaquinod M., Chroboczek J. (2003). Otubains: A new family of cysteine proteases in the ubiquitin pathway. EMBO Rep..

[23] Saldana M., VanderVorst K., Berg A.L., Lee H., Carraway K.L. (2019). Otubain 1: A non-canonical deubiquitinase with an emerging role in cancer. Endocr. Relat. Cancer.

[24] Juang Y.C., Landry M.C., Sanches M., Vittal V., Leung C.C., Ceccarelli D.F., Mateo A.R., Pruneda J.N., Mao D.Y., Szilard R.K., Orlicky S., Munro M., Brzovic P.S., Klevit R.E., Sicheri F. (2012). OTUB1 co-opts Lys48-linked ubiquitin recognition to suppress E2 enzyme function. Mol. Cell.

[25] Nakada S., Tai I., Panier S., Al-Hakim A., Iemura S., Juang Y.C., O'Donnell L., Kumakubo A., Munro M., Sicheri F., Gingras A.C., Natsume T., Suda T., Durocher D. (2010). Non-canonical inhibition of DNA damage-dependent ubiquitination by OTUB1. Nature.

[26] Messick T.E., Russell N.S., Iwata A.J., Sarachan K.L., Shiekhattar R., Shanks J.R., Reyes-Turcu F.E., Wilkinson K.D., Marmorstein R. (2008). Structural basis for ubiquitin recognition by the Otu1 ovarian tumor domain protein. J. Biol. Chem..

[27] Zhao L., Wang X., Yu Y., Deng L., Chen L., Peng X., Jiao C., Gao G., Tan X., Pan W., Ge X., Wang P. (2018). OTUB1 protein suppresses mTOR complex 1 (mTORC1) activity by deubiquitinating the mTORC1 inhibitor DEPTOR. J. Biol. Chem..

[28] Chang D.K., Ricciardiello L., Goel A., Chang C.L., Boland C.R. (2000). Steady-state regulation of the human DNA mismatch repair system. J. Biol. Chem..

[29] de Wind N., Dekker M., Claij N., Jansen L., van Klink Y., Radman M., Riggins G., van der Valk M., van't Wout K., te Riele H. (1999). HNPCC-like cancer predisposition in mice through simultaneous loss of Msh3 and Msh6 mismatch-repair protein functions. Nat. Genet..

[30] Marra G., Iaccarino I., Lettieri T., Roscilli G., Delmastro P., Jiricny J. (1998). Mismatch repair deficiency associated with overexpression of the MSH3 gene. Proc. Natl. Acad. Sci. U. S. A..

[31] Karunarathna U., Kongsema M., Zona S., Gong C., Cabrera E., Gomes A.R., Man E.P.S., Khongkow P., Tsang J.W.H., Khoo U.S., Medema R.H., Freire R., Lam E.W.F. (2016). OTUB1 inhibits the ubiquitination and degradation of FOXM1 in breast cancer and epirubicin resistance. Oncogene.

[32] Modrich P., Lahue R. (1996). Mismatch repair in replication fidelity, genetic recombination, and cancer biology. Annu. Rev. Biochem..

[33] Kaufmann S.H., Desnoyers S., Ottaviano Y., Davidson N.E., Poirier G.G. (1993). Specific proteolytic cleavage of poly(ADP-ribose) polymerase: An early marker of chemotherapy-induced apoptosis. Cancer Res..

[34] Tewari M., Quan L.T., O'Rourke K., Desnoyers S., Zeng Z., Beidler D.R., Poirier G.G., Salvesen G.S., Dixit V.M. (1995). Yama/CPP32 beta, a mammalian homolog of CED-3, is a CrmA-inhibitable protease that cleaves the death substrate poly(ADP-ribose) polymerase. Cell.

[35] Castri P., Lee Y.J., Ponzio T., Maric D., Spatz M., Bembry J., Hallenbeck J. (2014). Poly(ADP-ribose) polymerase-1 and its cleavage products differentially modulate cellular protection through NF-kappaB-dependent signaling. Biochim. Biophys. Acta.

[36] Kolodner R.D. (1995). Mismatch repair: Mechanisms and relationship to cancer susceptibility. Trends Biochem. Sci..

[37] Li G.M. (2013). Decoding the histone code: Role of H3K36me3 in mismatch repair and implications for cancer susceptibility and therapy. Cancer Res..

[38] Mao G., Lee S., Ortega J., Gu L., Li G.M. (2012). Modulation of microRNA processing by mismatch repair protein MutLalpha. Cell Res..

[39] Valeri N., Gasparini P., Braconi C., Paone A., Lovat F., Fabbri M., Sumani K.M., Alder H., Amadori D., Patel T., Nuovo G.J., Fishel R., Croce C.M. (2010). MicroRNA-21 induces resistance to 5-fluorouracil by down-regulating human DNA MutS homolog 2 (hMSH2). Proc. Natl. Acad. Sci. U. S. A..

[40] Valeri N., Gasparini P., Fabbri M., Braconi C., Veronese A., Lovat F., Adair B., Vannini I., Fanini F., Bottoni A., Costinean S., Sandhu S.K., Nuovo G.J., Alder H., Gafa R. (2010). Modulation of mismatch repair and genomic stability by miR-155. Proc. Natl. Acad. Sci. U. S. A..

[41] Fink D., Aebi S., Howell S.B. (1998). The role of DNA mismatch repair in drug resistance. Clin. Cancer Res..

[42] Le D.T., Durham J.N., Smith K.N., Wang H., Bartlett B.R., Aulakh L.K., Lu S., Kemberling H., Wilt C., Luber B.S., Wong F., Azad N.S., Rucki A.A., Laheru D., Donehower R. (2017). Mismatch repair deficiency predicts response of solid tumors to PD-1 blockade. Science.

[43] Le D.T., Uram J.N., Wang H., Bartlett B.R., Kemberling H., Eyring A.D., Skora A.D., Luber B.S., Azad N.S., Laheru D., Biedrzycki B., Donehower R.C., Zaheer A., Fisher G.A., Crocenzi T.S. (2015). PD-1 blockade in tumors with mismatch-repair deficiency. N. Engl. J. Med..

[44] Herhaus L., Perez-Oliva A.B., Cozza G., Gourlay R., Weidlich S., Campbell D.G., Pinna L.A., Sapkota G.P. (2015). Casein kinase 2 (CK2) phosphorylates the deubiquitylase OTUB1 at Ser16 to trigger its nuclear localization. Sci. Signal..

[45] Goldstein M., Kastan M.B. (2015). The DNA damage response: Implications for tumor responses to radiation and chemotherapy. Annu. Rev. Med..

[46] Morgan M.A., Lawrence T.S. (2015). Molecular pathways: Overcoming radiation resistance by targeting DNA damage response pathways. Clin. Cancer Res..

[47] Liu J., Kruswick A., Dang H., Tran A.D., Kwon S.M., Wang X.W., Oberdoerffer P. (2017). Ubiquitin-specific protease 21 stabilizes BRCA2 to control DNA repair and tumor growth. Nat. Commun..

