# Supplementary material for: OTUB1 stabilizes mismatch repair protein MSH2 by blocking ubiquitination
Source: J Biol Chem. 2021 Feb 26;296:100466. doi: 10.1016/j.jbc.2021.100466 (PMC8042173; doi:10.1016/j.jbc.2021.100466)
Supplement: Supplemental Figure S1 [file mmc1.pdf]

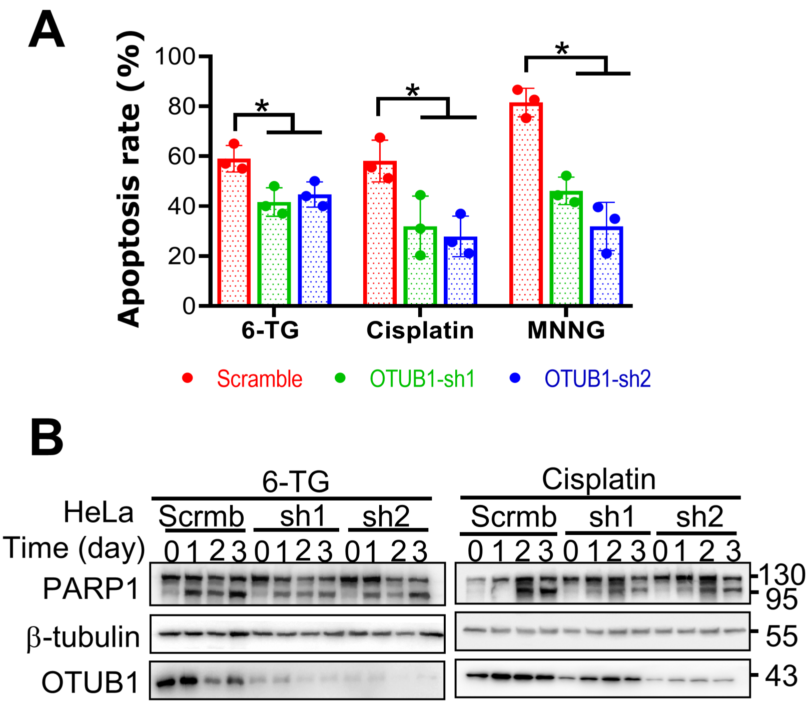

Fig. S1. A. Cell apoptosis analyses of OTUB1-KD (sh1 and sh2) and control (scrambled) HeLa cells treated with 6-thioguanine (6-TG), cisplatin or MNNG using FACS to detect AnnexinV and propidium iodide signal. B. Detection of cleaved PARP1 by Western blotting in OTUB1-KD and control HeLa cells treated with 6-TG or cisplatin for the indicated times.
